# Cerebrospinal fluid cytokines in geriatric patients with depressive disorders: A retrospective case-control study

**DOI:** 10.3389/fpsyt.2022.947605

**Published:** 2022-09-12

**Authors:** Morten Brix Schou, Jeanette Brun Larsen, Astrid Kamilla Stunes, Sverre Georg Sæther

**Affiliations:** ^1^Department of Mental Healthcare - General, Rehabilitation and Safety, St Olav's University Hospital, Trondheim, Norway; ^2^Department of Mental Health, Norwegian University of Science and Technology, Trondheim, Norway; ^3^Department of Clinical and Molecular Medicine, Faculty of Medicine and Health Sciences, Norwegian University of Science and Technology, Trondheim, Norway; ^4^Medical Clinic, St. Olavs University Hospital, Trondheim, Norway; ^5^Blue Cross Lade Addiction Treatment Centre, Trondheim, Norway

**Keywords:** neuroinflammation, cytokine—immunological terms, cerebrospinal fluid, affective disorders, depression

## Abstract

Central nervous system inflammation might play a role in patients with depressive disorders. This hypothesis is supported by studies reporting increased cerebrospinal fluid levels of the inflammatory markers interleukin (IL)-6, IL-8 and tumor necrosis factor alpha (TNF-α) in patients with ongoing depression. In this case-control study, we aimed to examine whether these findings also applied to depressed patients in a *geriatric population*. Cerebrospinal fluid cytokine analyses were performed on 15 patients (age >60 years) with depressive disorders and 45 age– and sex matched controls (patients with headache or idiopathic facial palsy). IL-6, IL-8, IL-10, TNF-α, monocyte chemoattractant protein-1 and transforming growth factor beta 1 were included in the statistical analyses. Patients with depression had significantly *lower* cerebrospinal fluid levels of IL-6 as compared to controls (*p* = 0.014) in the univariate analysis. The finding was, however, no longer statistically significant after correction for age and body mass index (*p* = 0.097). Overall, this study indicates that the cytokines included in this study are not significantly altered in geriatric patients with depression. Future studies exploring cerebrospinal fluid cytokine levels should include corrections for possible confounding factors.

## Introduction

There is robust evidence that depressive disorders are associated with elevated serum levels of a wide range of inflammatory markers, such as c-reactive protein and cytokines (1). In contrast to the overwhelming number of studies exploring the role of peripheral inflammation in depressive disorders, the literature on inflammatory markers in cerebrospinal fluid (CSF) is more limited, and in geriatric patients almost non-existing (2). The authors of a recent meta-analysis were able to detect three studies examining the CSF levels of cytokines in geriatric patients (age 60 years or older) with depressive disorders (2). These studies examined only three relevant inflammatory markers (interleukin (IL)-6, soluble-IL-6 receptor, and IL-8) and had contradictory findings. The authors of the first study found that depressive disorder was associated with *lower* CSF levels of IL-6 and soluble-IL-6 receptor (3). Using participants from the general population, the authors of a second study reported *higher* CSF levels of IL-6 and IL-8 in women with depression compared to those without depression (4). The authors of the third study found that patients with sole depressive disorder had *higher* CSF levels of IL-6 compared to patients with Parkinson's disease and comorbid depressive disorder (5).

In their meta-analysis, Enache et al. concluded that patients with depressive disorders have *increased* CSF levels of IL-6, IL-8 and Tumor necrosis factor alpha (TNF-α) (2), suggesting a possible inflammatory state. However, whether this is true for geriatric patients with depressive disorders remains unclear.

The primary aim of this study was to compare the CSF levels of IL-6, IL-8 and TNF-α in geriatric patients with depressive disorders and an age- and sex matched control group. Based on the evidence in younger patients with depression (2), we hypothesized that depressed geriatric patients had elevated levels of IL-6, IL-8 and TNF-α compared to controls. The secondary aim was to compare the levels of other relevant cytokines between the two groups, with the hypothesis that depressive disorders in geriatric patients are associated with increased CNS inflammation.

## Materials and methods

### Design and setting

This is a retrospective case-control study with patients and controls recruited from the Neurological Research Biobank at St. Olavs hospital, Trondheim University Hospital, Trondheim, Norway. All patients undergoing lumbar puncture at the neurologic department at St. Olavs hospital have since 2004 been asked for written informed consent to donate CSF for research purpose. For this specific study, patient inclusion ended the 31st of December 2016.

### Participants

Patients (cases) were eligible for inclusion in this study if they had a diagnosis of depressive disorder in their patient record at the time of the lumbar puncture [first episode (F32.x) or recurrent episode (F33.x) according to International Classification of Diseases version 10 (ICD-10)]. Controls were selected among patients referred to lumbar puncture because of headache or idiopathic facial palsy. Controls were age (±5 years)—and sex matched (3 controls per case) with the cases. Exclusion criteria for controls were a history of treatment in psychiatric specialist healthcare services and/or findings of an inflammatory condition (e.g., multiple sclerosis) after lumbar puncture.

### Demographic and clinical variables

Demographic and clinical variables such as age, sex, education, smoking, use of psychopharmacologic or anti-inflammatory medication, body mass index (BMI, kg·m^−2^) and primary diagnoses for the treatment contact were extracted from the patient charts.

### CSF cytokine analyses

CSF was collected by a standardized lumbar puncture procedure and stored at −80°C until thawed, centrifugedand aliquoted into smaller units in 2019, and further stored at −80°C until analyses in 2020. CSF were centrifuged immediately after thawing and before analyses for cytokines IL-1β, IL-2, IL-6, IL-8, IL-10, IL-17A, interferon (IFN)-γ, TNF-α, Monocyte Chemoattractant Protein-1 (MCP-1) by a MILLIPLEX® Magnetic Bead Panel CYTOMAG-60K-09 (Merck, Darmstadt, Germany) and Transforming growth factor beta 1 (TGF- β1) by a MILLIPLEX® Magnetic Bead Panel TGFBMAG-64K-03 (Merck), using a Bio-plex 2000 and the Bio-plex manager software (Biorad, Hercules, CA). Range of detected values and generation and validation of standard curves were performed for each setup. Standards and controls were diluted in a 1:40 ratio mix of standard diluent (containing 25% bovine serum) and sample diluent, to mimic a protein content close to CSF, and analyzed in duplicates for each plate setup. CFS samples were analyzed as undiluted in single samples. All analyses were performed according to the manufacturers' protocol. The range of detected values was as following: IL-1β: 0.02–0.58 pg/mL, IL-2: 0.02–0.53 pg/mL, IL-6: 0.22–26.62 pg/mL, IL-8: 18.32–187.15 pg/mL, IL-10: 0.11–5.23 pg/mL, IL-17A: 0.15–0.15 pg/mL, IFN- γ: 0.1–2.76 pg/mL, TNF-α: 0.04–6.75 pg/mL, MCP 1: 623.78–2692.63 pg/mL and TGF-β1 1.0–185.91 pg/mL. The number of samples and percentage under the detection limit was as follows: IL-1β 35 (58.3%), IL-2 38 (63.3%), IL-6 0 (0%), IL-8 0 (0%), IL-10 5 (8.3 %), IL-17A 59 (98.3%), IFN-γ 57 (95.0%), TNF-α 1 (1.7%), MCP-1 0 (0%) and TGF-β1 0 (0%). Cytokines with undetectable values in more than 30 % of patients were not further included in the analyses. Samples with cytokine levels below the detection limit were imputed with the lowest detectable value divided by 2 for further statistical analyses.

### Statistics

Differences in demographic and clinical data were tested with Student's *t*-test or Chi square test. Cytokine data were analyzed for using a Shapiro Wilk test and inspection of Q-Q plots. All cytokines except TGF-β1 were positively skewed. In order to achieve normal distribution, skewed variables were transformed either with square root, log or inverse transformation. In the control group, there were violations of normality for both IL-6 and IL-10 also after transformation of data. Regardless of this, further analyses were performed with Student's *t*-test for differences in cytokine levels between cases and controls. The data are presented as median and interquartile range (IQR) when not normally distributed, although the analyses were made with Student's *t*-test on transformed variables. A multiple linear regression model was used to further assess the difference of cytokine levels in cases and controls. Age and BMI were selected as covariates due to previous studies indicating that they affect cytokine levels (6–8). The level of significance was set at *p* ≤ 0.05, and all analyses were two-tailed. *P*-values were adjusted for multiple testing (Bonferroni correction) for the analyses of the primary aim. The analyses of the secondary aim were not corrected for multiple testing.

### Ethics

All participating patients gave written informed consent. The study was conducted in accordance with the Declaration of Helsinki and approved by The Regional Committee for Medical Research Ethics, Central Norway, REK number 064-04 and 2017/2377.

## Results

### Demographic and clinical variables

A total of 15 geriatric patients with depressive disorders were identified in the CSF biobank, and age– and sex matched with 45 controls. See Table 1 for demographic and clinical variables. There were a higher proportion of men among the cases than controls. This was due to a lack of elderly men available as controls in the CSF biobank. There was a higher proportion of smokers among cases compared to controls (*p* = 0.058). Nine out of 15 cases used psychopharmacological medications compared to three out of 45 of the controls. One case used prednisolone for polymyalgia rheumatica and was therefore excluded in the further analyses.

**Table 1:** Demographic and clinical characteristics of the study population.

	**Cases (*n =* 15)**	**Controls (*n =* 45)**	***p*-value**
Age, mean (SD)	71.2 (7.2)	68.2 (8.6)	0.23^a^
Sex, male, *n* (%)	6 (40)	12 (27)	0.33^b^
Education^c^, *n* (%)			0.50^b^
9 years	4 (33)	4 (18)	
12 years	2 (17)	9 (41)	
Bachelor	3 (25)	5 (23)	
Master or higher	3 (25)	4 (18)	
Smoking^d^, yes, *n* (%)	4 (27)	3 (8)	0.058^b^
BMI^e^, mean (SD)	24.6 (4.2)	25.6 (3.5)	0.42^a^
Diagnoses, *n* (%)			
Mild depression (F3x.0)	2 (13)		
Moderate depression (F3x.1)	6 (40)		
Severe depression (F3x.2)	3 (20)		
Severe depression with psychosis (F3x.3)	4 (27)		
Headache		31 (69)	
Bell's palsy		14 (31)	
Psychopharmacological medication^f^, *n* (%)	9 (60)	3 (7)	
Antidepressants	8 (53)	3 (7)	
Mood stabilizers^g^	3 (20)	0 (0)	
Antipsychotics	2 (13)	0 (0)	
Benzodiazepines	2 (13)	0 (0)	
Anti-inflammatory medication, *n* (%) (prednisolone, NSAIDs)	1 (7)	0 (0)	

a Student's t-test,

b Chi-square,

c Missing data on three cases and 23 controls,

d Missing data on five controls,

e Missing data on four cases and 13 controls,

f Four cases used medication from more than one medication category,

g Lithium (n = 2). Lamotrigine (n = 1).

BMI, Body Mass Index; SD, Standard Deviation; NSAIDs, Non-Steroidal Anti-Inflammatory Drugs.

### Cytokine levels

Patients with depressive disorders had significantly lower CSF levels of IL-6 as compared to controls (*p* = 0.014) in the univariate analysis, also after correction for multiple testing. There was a trend toward lower levels of IL-8 in cases compared to controls (*p* = 0.069). The cytokine levels in cases and controls are presented in Table 2. Since there was an overrepresentation of female participants, particularly in the control group, cytokines were also analyzed stratified on sex. These analyses showed no significant differences (see Supplementary Table 1). The difference in IL-6 levels was no longer statistically significant in a multiple linear regression model with cytokine levels as the dependent variable and age and BMI as covariates (*p* = 0.097). The regression analyses are summarized in Table 3. Supplementary Table 2 shows demographic and clinical parameters for cases (*n* = 11) and controls (*n* = 32) included in the regression model.

**Table 2:** CSF cytokine levels in cases and controls.

**Cytokine (pg/mL)**	**Cases (*n =* 14)**	**Controls (*n =* 45)**	***p*-value^a^**
IL-6, median (IQR)	0.50 (0.38–0.96)	0.88 (0.41–1.76)	0.014
IL-8, median (IQR)	26 (22–38)	32 (28–48)	0.069
TNF-α, median (IQR)	0.52 (0.24–0.92)	0.37 (0.24–0.69)	0.39
IL-10, median (IQR)	0.86 (0.39–1.27)	0.86 (0.44–1.27)	0.53
MCP-1, median (IQR)	1,144 (1,095–1,420)	1,283 (1,102–1,761)	0.40
TGF-β1, mean (SD)	118 (51)	112 (37)	0.61

a Student's t-test, IL: Interleukin, IQR; interquartile range, TNF-α; Tumor Necrosis Factor-α, MCP-1: Monocyte Chemoattractant Protein-1, TGF-β1: Transforming Growth Factor-β1, SD: Standard Deviation.

**Table 3:** Differences in cytokine levels between cases and controls after correction of confounding factors (age and body mass index).

**Cytokine**	**Cases (*n =* 11)^a^**	**Controls (*n =* 32)^a^**	***F***	***p*-value**
IL-6, mean (SEM)	−0.239 (−0.118–0.226)	0.054 (−0.538–0.059)	2.89	0.097
IL-8, mean (SEM)	0.034 (0.027–0.041)	0.030 (0.026–0.034)	0.75	0.39
TNF- α, mean (SEM)	−0.328 (-0.550–0.105)	−0.327 (−0.455–0.199)	0.00	1.0
IL-10, mean (SEM)	0.880 (0.632–1.128)	0.952 (0.810–1.095)	0.26	0.62
MCP-1, mean (SEM)	3.078 (2.993–3.162)	3.144 (3.095–3.192)	1.81	0.19
TGF-β1, mean (SEM)	112.8 (86.3–139.2)	107.7 (92.4–122.9)	0.11	0.74

SEM, standard error of mean; MCP, monocyte chemoattractant protein; IL, interleukin; TGF, transforming growth factor; TNF, tumor necrosis factor.

a Because BMI was missing on a number of cases and controls these analyses could only include 11 cases and 32 controls. See Supplementary Table S2 for demographic and clinical variables for this subset of participants.

None of the other cytokines differed significantly between the groups. Age was a significant covariate in the analyses of TNF-α and IL-10, while BMI did not show significance by itself in the linear regression model. The diagnosis, medication and cytokine levels of each case are presented in the Supplementary Table 3.

## Discussion

The main finding of this study is that CSF cytokine levels were not statistically significant different between patients with depression and controls after correction for confounding factors. Specifically, in this geriatric patient sample, we were not able to replicate previous findings of *elevated* CSF levels of inflammatory markers IL-6, IL-8 and TNF-α in patients with depressive disorders (2). In fact, the depressed patients had a significantly *lower* level of CSF IL-6 as compared to the controls in the univariate analyses. However, this finding was no longer significant after correcting for age and BMI.

Three previous studies have examined CSF levels of IL-6 in geriatric depressed patients. Kern et al. found *increased* CSF levels of IL-6 in depressed patients (4). However, the finding was no longer significant when they adjusted for smoking status and BMI using IL-6 as a continuous variable (the finding remained significant, however, when IL-6 was analyzed as a categorical variable based on quartiles). In other words, their finding might be in line with our study. Stübner et al. found *decreased* CSF levels of IL-6 in depressed patients (3). They found no correlation between IL-6 and age, but BMI was not assessed. Palhagen et al. found *lower* CSF levels of IL-6 in patients with sole depression as compared to patients with both Parkinson's disease and depression (5). The authors did not assess age and BMI, and the comparison with patients with Parkinson's disease limits the generalizability of the findings. Without the correction of BMI and age, these two studies are not directly comparable with our main finding.

There were no significant differences between groups in CSF levels of the other cytokines examined in this study. Kern et al. has previously reported increased levels of IL-8 in the CSF of geriatric depressed patients (4). We were not able to replicate this finding in our study. TNF-α and the other three cytokines examined in the present study has, to the best of our knowledge, never previously been assessed in the CSF of a geriatric depressed patient sample.

In previous research in patients with depression, serum cytokine levels have been more excessively examined than CSF cytokine levels. In a meta-analysis, the authors reported significant elevations of serum levels of several cytokines even after adjusting for confounding factors such as BMI, smoking status, and use of anti-depressive medications (1). However, the small number of CSF studies and the lack of assessment of confounding factors in these, make conclusions difficult to draw.

Enache et al. detected nine studies exploring CSF cytokine levels in depressed non-geriatric patients (2); three studies did not assess any confounding factor (9–11), four studies adjusted for one confounding factor (12–15) [smoking status (15), age (12, 14), BMI (13)], and one study examined correlations with age, BMI and smoking status (16). Two additional studies have been published since the meta-analysis was performed (17, 18). Kuzior et al. adjusted for sex and medication without it affecting their findings of increased IL-8 levels in depressed patients compared to patients with idiopathic intracranial hypertension. Hidese et al. did not find significant differences in cytokine levels between patients with depression and healthy controls. In this study, age and BMI correlated with cytokine levels in only a minor of the analyzed cytokines.

Age and BMI are important factors to address in studies exploring the role of peripheral and CNS inflammation in patients with mental disorders. Inflamm-aging is a term referring to the natural increase in low grade chronic inflammation with increasing age (19). This systemic inflammation is relevant for the development of several chronic disorders of the geriatric population. Similarly, BMI has been shown to correlate with peripheral inflammatory markers, such as proinflammatory cytokines (8). However, it is somewhat surprising that correction had an impact on the results in this study as both mean BMI and age were approximately equal in the groups. Still, with minor differences between cytokine levels in the unadjusted model, a small correction might be enough to make a finding non-significant. We argue that future studies should include correction of these confounding factors.

Several limitations of the present study must be considered. The number of included participants is low, which limits the possibilities to detect small but possibly relevant differences in cytokine levels.

Sixty percent of the cases used psychopharmacological medication at the time of lumbar puncture, most of them anti-depressive medication. Previous studies have shown that anti-depressive medication may be associated with a decrease in serum levels of pro-inflammatory cytokines (20, 21). In an inflammatory depression model in mice, anti-depressive medication prevented a lipopolysaccharide induced increase in CNS pro-inflammatory cytokines (22). Taken together, these studies open the possibility that the frequent use of anti-depressive medication among the patients in our study have precluded the detection of the hypothesized pro-inflammatory state. The controls used in this study were patients with headache (migraine or tension type) or idiopathic facial palsy. The authors of one previous study have reported higher CSF levels of MCP-1 and TGF-β in patients with migraine without aura and tension type headache compared to healthy controls (23). Even so, in this study by Bo and colleagues, they did not correct for possible confounding factors. Still, it is possible that both headache (24) and idiopathic facial palsy (25) are associated with inflammatory processes, limiting the chance of finding a difference in cytokine levels between our case and control group.

The study also withholds some strengths. Almost half of the patients had severe depression, indicating that we were able to include patients with prominent symptoms. All treatment in Norway is free of charge, and patients with various socioeconomic status were included. In addition to having controls matched on age and sex, we corrected for BMI and age in the statistical analyses.

The findings in this study do not support a role of CNS inflammation, as measured by altered CSF levels of IL-6, IL-8, TNF-α, IL-10, MCP-1 or TGF β, in geriatric patients with depressive disorders. The study highlights the importance of including possible confounding factors (such as age and BMI) in future studies exploring CSF cytokine levels in patients with depression.

## Data availability statement

The raw data supporting the conclusions of this article will be made available by the authors, without undue reservation.

## Ethics statement

The studies involving human participants were reviewed and approved by the Regional Committee for Medical Research Ethics, Central Norway. The patients/participants provided their written informed consent to participate in this study.

## Author contributions

MS and SGS designed the study. MS performed the statistical analyses and drafted the manuscript. MS, SGS, and JL collected clinical data. AS performed the laboratory analyses. All authors were involved in revising the manuscript for intellectual content and read and approved the final manuscript.

## Funding

The research was founded with a general grant from St Olavs Hospital.

## Conflict of interest

The authors declare that the research was conducted in the absence of any commercial or financial relationships that could be construed as a potential conflict of interest.

## Publisher's note

All claims expressed in this article are solely those of the authors and do not necessarily represent those of their affiliated organizations, or those of the publisher, the editors and the reviewers. Any product that may be evaluated in this article, or claim that may be made by its manufacturer, is not guaranteed or endorsed by the publisher.
