# Supplementary material for: Cerebrospinal fluid cytokines in geriatric patients with depressive disorders: A retrospective case-control study
Source: Front Psychiatry. 2022 Sep 12;13:947605. doi: 10.3389/fpsyt.2022.947605 (PMC9510363; doi:10.3389/fpsyt.2022.947605)
Supplement: Supplementary file 1 [file Data_Sheet_1.docx]

Supplementary Table 1 CSF cytokine levels in cases and controls stratified on sex.

| Male |  |  |  |
| --- | --- | --- | --- |
| Cytokine (pg/mL) | Cases (n=6) | Controls (n=12) | p - value^a^ |
| IL-6, median (IQR) | 0.65 (0.46-0.99) | 1.0 (0.66-1.83) | 0.082 |
| IL-8, median (IQR) | 26 (22-43) | 32 (26-56) | 0.39 |
| TNF-α, median (IQR) | 0.52 (0.24-0.92) | 0.37 (0.24-0.90) | 0.75 |
| IL-10, median (IQR) | 0.65 (0.39-1.27) | 1.27 (0.65-1.62) | 0.12 |
| MCP-1, median (IQR) | 1098 (996-1705) | 1464 (1133-1852) | 0.47 |
| TGF-β1, mean (SD) | 110 (62) | 112 (30) | 0.91 |
|  |  |  |  |
| Female |  |  |  |
| Cytokine (pg/mL) | Cases (n=8) | Controls (n=33) | p - value^a^ |
| IL-6, median (IQR) | 0.43 (0.34-0.92) | 0.66 (0.34-1.80) | 0.21 |
| IL-8, median (IQR) | 29 (23-38) | 34 (28-44) | 0.13 |
| TNF-α, median (IQR) | 0.55 (0.23-1.30) | 0.37 (0.25-0.62) | 0.46 |
| IL-10, median (IQR) | 0.91 (0.28-1.65) | 0.86 (0.44-1.06) | 0.97 |
| MCP-1, median (IQR) | 1170 (1138-1352) | 1244 (1095-1619) | 0.43 |
| TGF-β1, mean (SD) | 125 (44) | 112 (40) | 0.43 |

^a^Student’s t-test, IL: Interleukin, IQR; interquartile range, TNF-α; Tumor Necrosis Factor-α, MCP-1: Monocyte Chemoattractant Protein-1, TGF-β1: Transforming Growth Factor-β1, SD: Standard Deviation

Supplementary Table 2 Demographic and clinical characteristics of cases and controls included in regression analyses

|  | Cases  (n=11) | Controls (n=32) | p- value |
| --- | --- | --- | --- |
| Age, mean, (SD) | 71.7 (6.7) | 67.5 (7.6) | 0.11^a^ |
| Sex, male, n (%) | 3 (27) | 10 (31) | 0.80^b^ |
| Education^c^, n (%)  9 years  12 years  Bachelor  Master or higher | 3 (33)  2 (22)  3 (33)  1 (11) | 2 (13)  6 (38)  4 (25)  4 (25) | 0.50^b^ |
| Smoking^d^, yes n (%) | 2 (18) | 3 (10) | 0.48^b^ |
| BMI (mean (SD)) | 24.6 (4.2) | 25.6 (3.5) | 0.42^a^ |
| Diagnoses n (%)  Mild depression (F3x.0)  Moderate depression (F3x.1)  Severe depression F3x.2)  Severe depression with psychosis (F3x.3)  Headache  Bell’s palsy | 1 (9)  5 (45)  3 (27)  2 (18) | 23 (72)  9 (28) |  |
| Psychopharmacological medication^e^, n (%)  Antidepressants  Mood stabilizers^f^  Antipsychotics  Benzodiazepines | 7 (64)  6 (55)  2 (18)  2 (18)  1 (9) | 3 (9)  3 (9)  0 (0)  0 (0)  0 (0) |  |
| Anti-inflammatory medication, n (%) (prednisolone, NSAIDs) | 0 (0) | 0 (0) |  |

^a^ Student’s t-test, ^b^ Chi-square, ^c^ Missing data on two cases and 16 controls, ^d^ Missing data on two controls, ^e^ Three cases used medication from more than one medication category, ^f^ Lithium (n=1) Lamotrigine (n=1), BMI: Body Mass Index, SD: Standard Deviation, NSAIDs: Non-Steroidal Anti-Inflammatory Drugs.

Supplementary Table 3. Diagnosis, medication and cytokine levels in cases

| Case | Diagnosis (ICD-10) | Medication  (Generic name) | IL-6  (pg/mL) | IL-8  (pg/mL) | TNF-α (pg/mL) | IL-10 (pg/mL) | MCP-1 (pg/mL) | TGF-β1  (pg/mL) |
| --- | --- | --- | --- | --- | --- | --- | --- | --- |
| 1 | F33.1 |  | 1.05 | 44.5 | 1.55 | 1.87 | 1137.5 | 87.80 |
| 2 | F32.1 | mirtazapine | 0.75 | 19.2 | 6.75 | 1.77 | 1356.1 | 175.4 |
| 3 | F32.1 | venlafaxine, mirtazapine | 0.98 | 39.8 | 0.56 | 1.27 | 1609.8 | 103.5 |
| 4 | F33.0 | reboxetine, amitriptyline | 0.41 | 22.5 | 0.43 | 0.96 | 1338.2 | 125.4 |
| 5 | F33.1 | lithium, venlafaxine | 0.95 | 24.8 | 0.50 | 0.86 | 1926.2 | 1.0 |
| 6 | F33.2 | lamotrigine | 0.34 | 32.4 | 0.56 | 0.86 | 1196.4 | 177.0 |
| 7 | F33.2 |  | 0.27 | 31.2 | 0.40 | 0.44 | 1141.2 | 95.9 |
| 8 | F33.3 | olanzapine | 0.44 | 25.6 | 0.17 | 0.22 | 1108.5 | 64.4 |
| 9 | F32.3 | lithium, escitalopram | 0.39 | 21.9 | 0.24 | 0.44 | 1048.4 | 104.3 |
| 10 | F32.0 | citalopram, prednisolone | 0.27 | 21.8 | 0.17 | 0.44 | 1020.3 | 105.5 |
| 11 | F32.3 | escitalopram | 0.48 | 22.4 | 0.24 | 0.22 | 1631.4 | 185.9 |
| 12 | F33.3 |  | 1.09 | 59.8 | 0.53 | 1.27 | 838.9 | 121.3 |
| 13 | F32.2 |  | 0.77 | 37.7 | 1.06 | 1.27 | 1052.8 | 144.0 |
| 14 | F33.1 | citalopram, flupentixol | 0.34 | 25.9 | 0.04 | 0.06 | 1145.2 | 171.3 |
| 15 | F33.1 |  | 0.52 | 26.4 | 0.87 | 0.44 | 1143.7 | 104.2 |

ICD-10; International classification of diseases revision 10, IL: Interleukin, TNF-α; Tumor Necrosis Factor-α, MCP-1: Monocyte Chemoattractant Protein-1, TGF-β1: Transforming Growth Factor-β1
